# The use of core needle biopsy as first-line in diagnosis of thyroid nodules reduces false negative and inconclusive data reported by fine-needle aspiration

**DOI:** 10.1186/1477-7819-12-61

**Published:** 2014-03-24

**Authors:** Pierpaolo Trimboli, Naim Nasrollah, Leo Guidobaldi, Silvia Taccogna, Davide Domenico Cicciarella Modica, Stefano Amendola, Francesco Romanelli, Andrea Lenzi, Giuseppe Nigri, Marco Centanni, Luca Giovanella, Stefano Valabrega, Anna Crescenzi

**Affiliations:** 1 Section of Endocrinology and Diabetology, Ospedale Israelitico, Rome, Italy; 2 Section of Surgery, Ospedale Israelitico, Rome, Italy; 3 Section of Pathology, Ospedale Israelitico, Rome, Italy; 4 Department of Experimental Medicine, Sapienza University, Rome, Italy; 5 Department of Surgical and Medical Sciences, Sapienza University, Ospedale S. Andrea, Rome, Italy; 6 Department of Medico-Surgical Sciences and Biotechnologies, Sapienza University, Latina, Italy; 7 Department of Nuclear Medicine and Thyroid Center, Oncology Institute of Southern Switzerland, Bellinzona, Switzerland

**Keywords:** Thyroid cancer, Core needle biopsy (CNB), Ultrasonography, Fine-needle aspiration (FNA)

## Abstract

**Background:**

The reported reliability of core needle biopsy (CNB) is high in assessing thyroid nodules after inconclusive fine-needle aspiration (FNA) attempts. However, first-line use of CNB for nodules considered at risk by ultrasonography (US) has yet to be studied. The aim of this study were: 1) to evaluate the potential merit of using CNB first-line instead of conventional FNA in thyroid nodules with suspicious ultrasonographic features; 2) to compare CNB and FNA as a first-line diagnostic procedure in thyroid lesions at higher risk of cancer.

**Methods:**

Seventy-seven patients with a suspicious-appearing, recently discovered solid thyroid nodule were initially enrolled as study participants. No patients had undergone prior thyroid fine-needle aspiration/biopsy. Based on study design, all patients were proposed to undergo CNB as first-line diagnostic aspiration, while those patients refusing to do so underwent conventional FNA.

**Results:**

Five patients refused the study, and a total of 31 and 41 thyroid nodules were subjected to CNB and FNA, respectively. At follow-up, the overall rate of malignancy was of 80% (CNB, 77%; FNA, 83%). However, the diagnostic accuracy of CNB (97%) was significantly (*P* < 0.05) higher than that of FNA (78%). In one benign lesion, CNB was inconclusive. Four (12%) of the 34 cancers of the FNA group were not initially diagnosed because of false negative (N = 1), indeterminate (N = 2) or not adequate (N = 1) samples.

**Conclusions:**

CNB can reduce the false negative and inconclusive results of conventional FNA and should be considered a first-line method in assessing solid thyroid nodules at high risk of malignancy.

## Background

Ultrasound (US) examination is the standard method for stratifying risk for malignancy in both palpable and non-palpable thyroid lesions, thus determining the need for fine-needle aspiration (FNA) [1,2]. A number of publications attest to the specific features by US and color flow Doppler (CFD) that are suggestive of malignancy, namely hypoechogenicity, irregular or blurred margins, microcalcifications, taller shape, and vascular signals [3,4]. In addition to conventional US, elastography is believed to improve the management of thyroid lesions, increasing the sensitivity of US-CFD when done in tandem [5]. As a consequence, cold nodules with the above features qualify for biopsy [1,2].

Although FNA can accurately distinguish thyroid cancers from benign lesions, there are a number of limitations [1,2]. The chief drawback is that up to 25 to 30% of cytologic samplings are inconclusive (that is, indeterminate or inadequate), often necessitating diagnostic surgery [1,2]. A low percentage of these ultimately prove malignant by histology, thus a means to further differentiate benign and cancerous lesions is advantageous. Moreover, many sources indicate that the falsenegative rate of FNA cytology with respect to thyroid cancers is not insignificant [6-8]. However, thyroid core needle biopsy (CNB) has recently been cited as a reliable test [9-14]. The microhistology of CNB samplings enables diagnosis in a large percentage of thyroid lesions deemed indeterminate or inadequate by FNA. The semi-automated CNB needles used are of small caliber (20 to 22 gauge), allowing full access to both large and small thyroid nodules with few complications and high patient tolerability [15]. Although the popularity of CNB has risen worldwide, this technique has primarily assumed a lesser role in thyroid lesions diagnosis, reserved for instances of inconclusive cytology. There are no data on its use as a first-line diagnostic tool in thyroid nodules at high risk of malignancy.

This study was designed to evaluate the potential merit of using CNB first-line instead of conventional FNA to assess thyroid nodules with suspicious US features. Thyroid nodule diagnostic results with CNB were compared with outcomes of patients subjected to FNA.

## Methods

### Patients

From September 2012 to April 2013, all patients referring to Ospedale Israelitico of Rome with a recently discovered suspicious thyroid nodule were selected for this study. None of the patients had undergone prior thyroid nodule aspiration/biopsy. All nodules were found to be solid at US and hot lesions were excluded. Seventy-seven patients (females, 64; males, 13) granting informed consent were initially enrolled as study participants. The study design proposed that all patients undergo CNB as first-line diagnostic aspiration, and those patients refusing CNB were tested by conventional FNA.

### Ultrasound examination

As in an earlier prospective multicenter study of ours [5], US risk stratification was aligned with Society of Radiologists in Ultrasound [16] and AACE/AME/ETA criteria [1]. The pattern of vascular signals within nodules was scrutinized by CFD and designated as absent, perinodular, or intranodular [17]. Real-time elastography (RTE) was also performed, ranking nodules as class I (prevalence of red and green color), class II (green with prevalence in more than 50% of the nodule), class III (blue in at least 50% of nodule), or class IV (blue with prevalence in at least 75% of nodule) [5]. To qualify as an ‘at-risk’ nodule by US examination, at least two of the following features were required: marked hypoechogenicity, microcalcifications, irregular margins, taller rather than wider shape, intranodular vascularization, and RTE class III to IV.

In all patients, US, CFD, and RTE examinations were performed prior to aspiration by an experienced endocrinologist, using an EsaoteMyLab system (Esaote, Genova, Italy) with linear probe. Ultrasonographic evaluation of each nodule was repeated by a second expert examiner. Any discordant cases were mutually resolved by the two physicians for definitive classification. Before the study began, US technique was standardized by the two operators for procedural reproducibility [5].

### FNA and CNB procedures

Patients were instructed to refrain from anticoagulants during the five days prior to FNA and CNB. Both biopsies were performed at Ospedale Israelitico of Rome by the same experienced surgeon in freehand fashion under US guidance. For FNA, a 23-gauge needle was utilized, while for CNB a 21-gauge Menghini cutting needle (Biomol, Hospital Service, Rome, Italy) was employed, as previously described [14,15]. Two needle passes were made for both biopsies. Before CNB, a local anesthetic was administered.

### Cytologic and microhistologic examination

Cytologic specimens were screened by an expert cytopathologist, conforming to AACE/AME/ETA [1] as follows: class 1 (inadequate), class 2 (benign), class 3 (indeterminate), class 4 (suspicious of malignancy), or class 5 (malignant).

Formalin-fixed needle cores underwent automated processing for embedding in paraffin, as we previously detailed [14,15]. Serial sections at 4 μm were affixed to polarized slides. A single H&E-stained slide was generated, and the remainder were immunostained for galectin-3, cytokeratin-19 and HBME-1 (Thermo Fisher Scientific, Fremont, CA, USA), using a biotin-free peroxidase method. All specimens were evaluated by an experienced pathologist.

All cytologic and microhistologic samples were also reviewed by a second pathologist. In case of disagreement, definitive reporting was achieved by mutual consensus.

### Follow-up

Final histologic examination was the ‘gold standard’ in all patients. Thyroid tumor classification relied on the most recent World Health Organization histologic criteria [18].

### Statistical analysis

Comparison of frequency was made by chi-squared test or Fisher exact test, as appropriate. Means and standard deviations were compared by the Student’s t-test. Statistical analysis was performed using Graph Pad Prism (Graph Pad Software Inc, La Jolla, CA, USA).

## Results

Five patients declined to participate in the study, and the final series included 72 nodules (size 11.5 ± 4.8 mm) from 72 patients (females, 60; males, 12; mean age, 50.4 ± 13.7 years). A proportion of these patients consented to undergo CNB (N = 31), while the remainder refused CNB and were tested via routine FNA (N = 41). Table 1 details the US nodules characteristics of the two groups.

**Table 1:** Main ultrasound characteristics of 72 solid nodules included in the study

	**CNB group**	**FNA group**	***P***
Size (mm)	12.3 ± 3.8	11.2 ± 5.1	0.31
Marked hypoechogenicity	26 (82.9%)	37 (90.2%)	0.48
Taller shape	5 (16.1%)	7 (17.1%)	0.83
Microcalcifications	14 (45.2%)	21 (51.2%)	0.78
Intranodular vascularization	17 (54.8%)	20 (48.8%)	0.78
Score III to IV at elastography	26 (83.9%)	32 (78.0%)	0.75
More than two risk features	15 (48.4%)	17 (41.5%)	0.72

Legend: CNB, core needle biopsy; FNA, fine-needle aspiration. Marked hypoechogenicity, solid hypoechoic nodule with no cystic areas. Taller shape, taller than wide shaped nodule. Microcalcifications, hyperechoic punctuations <2 mm. Intranodular vascularization, intralesional vascular signal. Score III to IV at elastography, blue color in more than 50% of the nodule.

### CNB group

**Figure 1:**
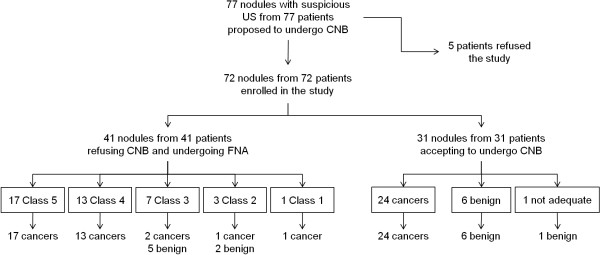
Study design and main results.

Mean size of the 31 nodules subjected to CNB was 12.3 ± 3.8 mm. Of these, 30 (96.8%) were diagnostic: 24 cancers and 6 benign lesions. One nodule (3.2%) was found to be inadequate. The histologic examination confirmed cancer in all 24 cases (classic papillary cancers, 14; follicular variant of papillary cancer, 9; medullary carcinoma, 1) and benign lesions in the remaining 6 specimens (nodular thyroiditis, 4; microfollicular hyperplasia, 2). The inadequate CNB sampling proved to be microfollicular hyperplasia (Figure 1).

### FNA group

**Figure 2:**
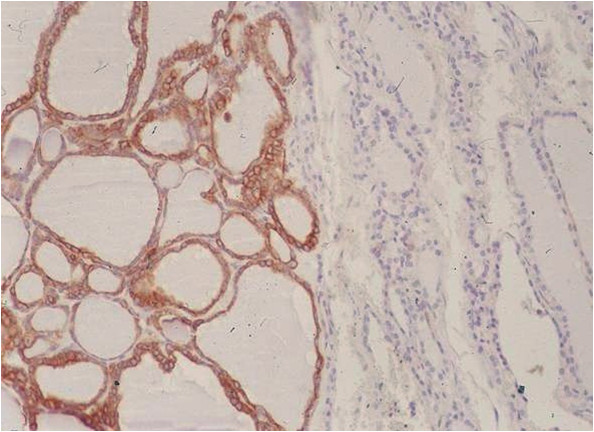
**Follicular variant of papillary carcinoma (immunohistochemistry for CK19).** Note the macrofollicular pattern of the papillary neoplasm.

Of the 41 nodules (mean size 11.2 ± 5.1 mm) subjected to FNA, 33 (80.5%) were initially diagnostic (class 5, 17; class 4, 13; class 2, 3). The other 8 (19.5%) were mostly indeterminate, with the exception of one inadequate specimen (Figure 1). Histologic confirmation of cancer was obtained for all patients with class 4 or 5 cytology. FNA was repeated in patients with class 2: 2 nodules were benign (class 2) with microfollicular hyperplasia at histology, while the other one was cancer (class 5) with postsurgical follicular variant of papillary carcinoma (Figure 2). The eight nodules with inconclusive (class 1 or class 3) results by FNA were selected for complementary CNB sampling. Of these, five were benign lesions (nodular thyroiditis, four; microfollicular hyperplasia, one) and three were considered malignant, as confirmed after surgery. Histologic features of 34 cancers from the FNA group are shown in Table 2.

**Table 2:** Final histologic diagnosis of 34 cancers from fine-needle aspiration (FNA) group

**Cytologic class**	**Cases (n)**	**Histology**
Class 1	1	Follicular variant of papillary carcinoma
Class 2	1	Follicular variant of papillary carcinoma
Class 3	2	Hürthle cell carcinoma (n = 1)
		Follicular variant of papillary carcinoma (n = 1)
Class 4	13	Classic papillary carcinoma (n = 4)
		Follicular variant of papillary carcinoma (n = 7)
		Hürthle cell carcinoma (n = 2)
Class 5	17	Classic papillary carcinoma (n = 13)
		Follicular variant of papillary carcinoma (n = 3)
		Columnar variant of papillary carcinoma (n = 1)

Legend: class 1, inadequate;class 2, benign;class 3, indeterminate;class 4, suspicious for malignancy;class 5, malignant.

### Comparison of the results from the two groups

No significant difference was found in nodule size and US characteristics between the two groups (Table 1). At follow-up, the overall rate of malignancy in the 72 patients was 80.5% (CNB, 77.4%; FNA, 82.9%). Diagnostic accuracy was significantly (*P* < 0.05) higher in CNB (30/31; 96.8%) than in FNA (32/41, 78%) group. No false negatives were recorded in CNB samples. In the FNA series, 4 (12%) of the 34 cancers were not initially diagnosed (false negative, 1; indeterminate, 2; not adequate, 1) and a 2.5% rate of false negative (class 2) results was recorded (Figure 1). Similar to our previous experiences [15,16], no complications were recorded after the biopsies.

## Discussion

Ultrasonographic risk stratification is the standard means of determining the need for biopsy in patients with thyroid nodules. Although FNA is the most cost-effective method of thyroid biopsy at present [1], a considerable percentage of cytologic preparations are diagnostically inconclusive. Molecular or genetic markers and clinical or instrumental features can be used to bolster FNA outcomes [19-24] but diagnostic surgery is often unavoidable [1,2,24]. CNB was introduced to better assess these thyroid nodules, and this approach is gaining momentum [9-14]. Unfortunately, its role has been limited to a complementary use in patients with inconclusive FNA results. Data from previous studies indicate that up to 98% of nodules indeterminate by FNA are diagnosable by CNB, and the combination of CNB and second-round FNA identifies 97% of nodules with prior inadequate FNA [9-14]. Therefore, chances of tailoring the surgical approach and reducing unnecessary surgery are substantial [25].

In the present study, our aim was to investigate the potential role of CNB as a first-line technique instead of conventional FNA for assessing thyroid nodules at risk of cancer by ultrasonography. To date, this particular approach has not been studied. It is generally accepted that the likelihood of malignancy rises if risk features are identified in a thyroid nodule by US [1,2], and that FNA should be repeated under these circumstances, regardless of a benign cytologic result [6-8]. Recently, the diagnostic utility of CNB was explored in sonographically suspicious thyroid nodules with initially benign cytologies, exposing a 32% rate of cancer [26]; in agreement with previous reports [9-15], CNB provided reliable diagnostic accuracy, reducing the need for repetitive FNA or diagnostic surgery.

Herein, we selected 72 patients with suspicious thyroid nodules for either CNB or FNA as the first-line diagnostic procedure, comparing subsequent results. An 80% malignancy rate was recorded because patients were at risk for cancer. Ultimately, CNB (97%) significantly outperformed FNA (78%) in terms of diagnostic accuracy. FNA results also failed to reflect four cancers with a 2.5% rate of false negatives (class 2), and five other benign lesions were cytologically indeterminate. Furthermore, there were no false negatives by CNB, and only one benign nodule was inadequate at CNB sampling.

Thyroid cytology is recognized as accurate, but its limitations with respect to misdiagnosis must be acknowledged. Aside from a certain percentage of inconclusive diagnoses by cytology demonstrated here (8/41, 19%), the potential for false results also exists. False negatives may be due poorly cellular aspirates or sampling error [27], approaching 11% in large and cystic lesions [28]. Similarly, papillary cancer may be misclassified as benign, owing to macrofollicular presentation in the absence of tell-tale cytologic alterations and architectural atypia (microfollicular or papillary pattern). In a recent paper, most false negatives were attributable to the follicular variant of papillary cancer [29]. Importantly, it must be emphasized that cancers not easily detected by FNA have higher rates of vascular and capsular invasion, increasing the odds of persistent disease [30]. On the other hand, the cutting needles of CNB enable microhistologic sampling of both large and small size thyroid nodules, with a high degree of accuracy. Expanding on our previous reports [9-14,26], we have shown for the first time that CNB should be considered the first-line approach in nodules defined at-risk by US to avoid falsenegative results and to reduce the reports found to beinconclusive by FNA.

Furthermore, patient tolerability of CNB during this study was excellent, as we previously reported [15]. In addition, responses to a questionnaire evaluating patient comfort level confirmed previous findings (data not shown).

For clinical practice, based on our previous experience and according to the present data, we were discouragedfrom repeating FNA after an initial inconclusive cytologic report. In this circumstance, as well as in the occurrence of recently discovered thyroid nodules defined as ‘suspicious for malignancy’, CNB should be used. This approach could avoid the ‘risk’ of false negative FNA and a delay in diagnosing thyroid cancers.

## Conclusions

In conclusion, our findings indicate a higher rate of diagnostic accuracy with first-line use of CNB than FNA for assessing at-risk thyroid nodules identified by US. The false negatives and inconclusive results of FNA may also be reduced making CNB the preferred method in this setting.

## Abbreviations

CFD: color flow Doppler; CNB: core needle biopsy; FNA: fine-needle aspiration; RTE: real-time elastography; US: ultrasonography.

## Competing interests

The authors declare that they have no competing interests.

## Authors’ contributions

PT, GN, NN, SA, ST, DDCM: acquisition of data, drafting of manuscript; PT, GN, SV, MC: study conception and design; PT, NN: performed ultrasound examination; NN: performed FNA and CNB; AC, ST, LeGu: analysis and interpretation of FNA and CNB data; AL, FR, LuGi: critical revision and supervision. All authors read and approved the final manuscript.
